# Huanglian Jiedu Decoction Exerts Antipyretic Effect by Inhibiting MAPK Signaling Pathway

**DOI:** 10.1155/2021/2209574

**Published:** 2021-12-31

**Authors:** Xing Li, Shizhang Wei, Xiao Ma, Haotian Li, Manyi Jing, Honghong Liu, Shengqi Niu, Yuling Tong, Lisheng Chen, Ying Wei, Sichen Ren, Yanling Zhao

**Affiliations:** ^1^School of Traditional Chinese Medicine, Southern Medical University, Guangzhou, China; ^2^Department of Pharmacy, The Fifth Medical Center of Chinese PLA General Hospital, Beijing, China; ^3^College of Pharmacy, Chengdu University of Traditional Chinese Medicine, Chengdu, China; ^4^Integrated TCM and Western Medicine Department, The Fifth Medical Center of Chinese PLA General Hospital, Beijing, China; ^5^Department of Pharmacy, Medical Supplies Centre of PLA General Hospital, Beijing, China; ^6^Department of Pharmacy, Hebei North University, Zhangjiakou, China

## Abstract

**Aim:**

The aim of this study was to explore the antipyretic effect and potential mechanism of Huanglian Jiedu Decoction (HLJDD) on LPS-induced fever in rats.

**Materials and Methods:**

The fever rat model was established by LPS. Anal temperature of rats was measured every 1 hour after modeling. TNF-*α*, IL-6, PGE_2_, and cAMP in rat serum or hypothalamus tissue were detected by ELISA kit. In order to explore the potential active ingredients and mechanism of antipyretic effect of HLJDD, we predicted the underlying antipyretic mechanism by using network pharmacology and then verified its mechanism by Western Blotting.

**Results:**

The results showed that HLJDD can alleviate LPS-induced fever in rats. The expression levels of TNF-*α*, IL-6, PGE_2_, and cAMP in the treatment group were significantly lower than those in the model group. Western Blotting results showed that the protein expression of p-ERK, p-JNK, and p-P38 was significantly inhibited.

**Conclusion:**

The findings suggest that HLJDD has a good antipyretic effect on LPS-induced fever in rats, which may be closely related to the inhibition of MAPK signaling pathway.

## 1. Introduction

Fever is a complex physiological stress response characterized by a regulatory rise in body temperature in response to inflammation or infectious disease [1]. Normally, the body maintains a dynamic balance between heat production and heat loss. When this balance is upset, the body temperature becomes abnormal. Fever is a controlled increase in body temperature, a hypothalamic-mediated response caused by pathogenic injury or invasion [2, 3]. This reaction promotes the synthesis of endogenous heat-producing factors, such as TNF-*α*, IL-6, PGE_2_, and cAMP, which will cause a series of biochemical and physiological changes in the body and eventually lead to elevated body temperature [4–7]. Hyperthermia is a treatment method that heats the temperature of a specific part of the body or the whole body to above the normal body temperature, so as to achieve the therapeutic effect. Traditional Chinese medicine (TCM) has heat therapy, such as sweat steaming, moxibustion, and cupping. Modern research shows that the combination of hyperthermia and chemotherapy is more effective than chemotherapy alone in the treatment of cancer diseases [8]. Studies have also found that increased body temperature regulation may be beneficial to the improvement of human immunity and reduce the sensitivity to infectious diseases [9]. However, in addition to being good for the body, fever can also be harmful [10]. For example, uncontrolled fever is associated with worse outcomes in patients with sepsis or neuronal damage [11].

TCM is a great treasure house, and it has unique advantages in the treatment of fever diseases. Chinese medicine classifies fever into two categories: fever due to external sensation and fever due to internal injury. External fever is caused by the feeling of external evil. Internal fever is caused by the imbalance of Yin, Yang, Qi, and blood in the internal organs. In external fever, the onset is rapid and the duration is short. In internal fever, the onset is slow and the duration is long, from weeks and months to years. According to the principles of TCM diagnosis and treatment, TCM treatment of fever includes the method of relieving symptoms and reducing fever, the method of dispelling dampness and reducing fever, and the method of nourishing Yin and reducing fever. Huanglian Jiedu Decoction (HLJDD), which originated from *Medical Secrets of an Official* (*Wai Tai Mi Yao* as named in Chinese) in the Tang Dynasty, was created by Tao Wang, a famous medical scientist in the Tang Dynasty. The original prescription of HLJDD is composed of *Coptidis Rhizoma*, *Scutellariae Radix*, *Phellodendri Chinensis Cortex*, and *Gardeniae Fructus* in the ratio of 3 : 2 : 2 : 3, which has the effect of clearing heat and removing toxicity. Clinical evidence suggests that HLJDD can be used in the treatment of a variety of diseases, which can relieve symptoms with a good clinical effect [12]. Pharmacological studies show that HLJDD has significant anti-inflammatory, antibacterial, and antiendotoxin activities [13, 14]. It can be used to treat high fever in children [15], sepsis, and other diseases [16].

Lipopolysaccharide (LPS), commonly known as endotoxin, is an important cell wall component of Gram-negative bacteria [17]. The proinflammatory effects of LPS play an important role in inhibiting bacterial infection. However, dysregulation of the host response to LPS may lead to systemic inflammation, such as sepsis [18]. In recent years, LPS is often used to establish animal models of fever [19–21]. Studies have shown that MAPK signaling pathway plays a key role in regulation of the production of proinflammatory mediators in LPS-induced inflammatory responses [22–24]. However, the effects of HLJDD in LPS-induced fever and the relationship between HLJDD and MAPK signaling pathways are still unclear.

TCM has multicomponent, multitarget, and multipathway characteristics. The therapeutic mechanisms and material basis of many herbal medicines have not been elucidated. With the rapid development of bioinformatics and various medical databases, network pharmacology has strongly contributed to the understanding of the molecular mechanisms of TCM from a holistic and systemic perspective [25]. Meanwhile, it has great advantages in predicting the target of TCM components, discovering multitarget drugs and providing new insights for the study of TCM [26]. HLJDD is a classical Chinese medicine formula for relieving fever and is also commonly used in Chinese medicine clinics. Its antipyretic effect is remarkable, but its antipyretic mechanism has not been completely elucidated. In order to initially explore this problem, this study was conducted to comprehensively evaluate the antipyretic effect of HLJDD by establishing an animal model of fever, combined with modern bioinformatics technology, aiming to provide ideas for the development of new drugs for the efficient, safe, and rapid treatment of fever symptoms. Meanwhile, it aimed to provide reasonable dosing guidance and lay an experimental foundation for the clinical application of HLJDD.

## 2. Materials and Methods

### 2.1. Drugs and Reagents

*Coptidis Rhizoma* (batch number: 20121701), *Scutellariae Radix* (batch number: 19101501), *Phellodendri Chinensis Cortex* (batch number: 19120601), and *Gardeniae Fructus* (batch number: 18091101) were purchased from Beijing Lvye Pharmaceutical Co., Ltd. (Beijing, China), and chemically authenticated by thin layer chromatography (TLC) in accordance with the instructions of Chinese Pharmacopoeia. The content was determined by high performance liquid chromatography (HPLC). The product inspection report numbers are CP-20-12-20, CP-19-10-07, CP-19-12-05, and CP-18-09-09, respectively. All detection results show that the quality of *Coptidis Rhizoma*, *Scutellariae Radix*, *Phellodendri Chinensis Cortex*, and *Gardeniae Fructus* is in full compliance with the regulations of the Chinese Pharmacopoeia version 2015. LPS from *Escherichia coli* 0111:B4 purified by phenol extraction was purchased from Sigma (batch number: 000010267).

### 2.2. Preparation of HLJDD

Weigh *Coptidis Rhizoma*, *Scutellariae Radix*, *Phellodendri Chinensis Cortex*, and *Gardeniae Fructus* at 3 : 2 : 2 : 3 and soak 30 min in pure water (1/10, w/v). Then it was extracted twice by heating (1 h at a time) [27]. After filtering and drying, the yield of HLJDD was 17.90%.

### 2.3. Ethics Statement

This study was carried out in accordance with the recommendations of the Guidelines for the Care and Use of Laboratory Animals of the Ministry of Science and Technology of China. All operations related to animal experiment are examined and approved by the Animal Ethics and Experimental Committee of the Fifth Medical Center of the PLA General Hospital (Approval ID: IACUC-2019-004).

### 2.4. Animals

50 male Sprague-Dawley rats (weighing 180 g–200 g) were purchased from Spiffy Biotechnology Co., Ltd. (Beijing, China, Permission No. SCXK-(Jing) 2019-0010). They were fed adaptively for 7 days under the conditions of temperature 25 ± 0.5°C, relative humidity 55 ± 5%, and alternating light (12 h light/dark cycle) and with free access to sufficient food and water. The anal temperature of rats was measured for 3 consecutive days, and the average value was taken as the baseline temperature of rats.

### 2.5. Establishment of LPS-Induced Fever Model and Drug Administration

The animals were randomly divided into 5 groups (*n* = 10), including control group, LPS-induced fever model group (100 *μ*g/kg, i.p.), HLJDD low dose group (1.58 g/kg), HLJDD medium dose group (3.15 g/kg), and HLJDD high dose group (6.30 g/kg). The administered medium dose of HLJDD was determined based on the recommended human dose (30 g/60 kg/day). The HLJDD groups were orally given different doses of HLJDD 1 h before the injection of LPS. At the same time, the control group were orally given with the same volume of normal saline. 1 hour after drug administration, the model group and each HLJDD group were intraperitoneally injected LPS (100 *μ*g/kg) to establish fever rat model. Then the anal temperature of the rats was measured every 1 hour. The last anal temperature was measured at 7 hours, the rats were euthanized and blood was taken through the abdominal aorta, and then the hypothalamus tissue was collected for subsequent studies.

### 2.6. Measurement of the Serum and Hypothalamus Tissue Levels of Biochemical Indexes

The ELISA kit (enzyme linked biology, Shanghai, China) was used for the detection of serum or hypothalamus tissue levels of TNF-*α*, IL-6, PGE_2_, and cAMP in accordance with the manufacturer's instructions.

### 2.7. Mechanism Prediction of HLJDD against Fever

#### 2.7.1. Identification Targets of HLJDD and Fever

Traditional Chinese Medicine Systems Pharmacology Database (TCMSP, https://old.tcmsp-e.com/tcmsp.php) was searched to collect the chemical components of the four herbs contained in HLJDD. The screening strategy used for potential active ingredients was oral bioavailability (OB) ≥30% and drug-likeness (DL) ≥0.18. Fever targets were collected from the GeneCard database (https://www.genecards.org/). The intersection targets of HLJDD and fever were obtained from Venn database (http://www.bioinformatics.com.cn/static/others/jvenn/example.html).

#### 2.7.2. Construction of Compound-Target Network

After the components and their corresponding target data were collected, the chemical components and potential targets of the abovementioned drugs in HLJDD were uploaded to Cytoscape3.8.0 to construct the Compound-Target network.

#### 2.7.3. Construction and Analysis of PPI Network

The intersection targets of HLJDD components and fever were uploaded to STRING database (https://string-db.org/), and the screening conditions were with confidence score ≥0.7, so as to establish PPI interaction network. Then, the interacting proteins screened by STRING were imported into Cytoscape3.8.0 to collect the topological parameter values of each interaction target in PPI network, and then the core targets were screened and collected.

#### 2.7.4. GO Enrichment and KEGG Pathway Analysis

The database (DAVID) (https://david.ncifcrf.gov/) has annotation, visualization, and integrated discovery capabilities. We therefore applied the DAVID database for gene ontology (GO) analysis and Kyoto Encyclopedia of Genes and Genomes (KEGG) pathway enrichment analysis.

### 2.8. Experimental Verification on the Mechanism of HLJDD against LPS-Induced Fever

#### 2.8.1. Western Blotting Analysis for Protein Expression

The total protein in the hypothalamus tissue was extracted by using high-efficiency RIPA lysis buffer supplemented with protease inhibitor and phosphatase inhibitor. The hypothalamus tissue homogenate was centrifuged at 4°C and 12000 g for 10 min. Protein concentration was determined by BCA Protein Assay Kit (Solarbio, Beijing, China). The protein samples were separated by 10% SDS-PAGE of gel at 80 V for 30 min and 120 V for 1 h and then transferred to polyvinylidene fluoride (PVDF) membrane (Millipore, MA, USA). After transmembrane, the PVDF membrane was removed and placed in Tris-buffered saline (TBS) containing 5% skim milk powder, which was sealed at room temperature for 2 h, and then incubated with primary antibodies at 4°C overnight; the detailed information is as illustrated in Table 1. After primary antibody incubation, wash with TBS-0.1% Tween 20 (TBST) buffer solution at room temperature for 3 times, 5 min each time. Then the PVDF membrane was placed into the secondary antibody solution and incubated at room temperature for 1 h. GAPDH was used as internal reference. ImageJ software was used for quantitative analysis.

### 2.9. Statistics Analysis

All data was presented as mean ± standard deviation (‾*X* ± SD). The differences between the group means were calculated by one-way ANOVA and Duncan's multirange test with the SPSS computer program (version 26.0). GraphPad Prism software (version 8.2.1) was used to visualize the results. *P* < 0.05 was considered statistically significant and *P* < 0.01 was highly significant.

## 3. Results

### 3.1. Effect of HLJDD on LPS-Induced Fever in Rats

**Figure 1:**
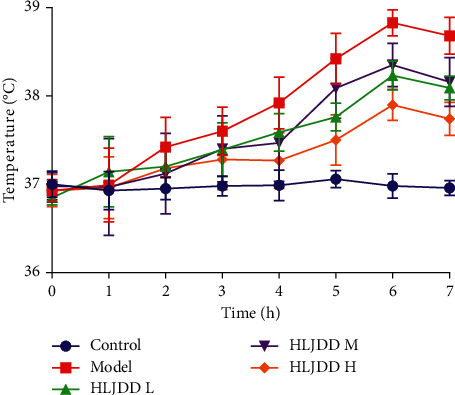
Changes of anal temperature in each group. The temperature at 0 h was the baseline temperature of rats, and the anal temperature was measured every 1 hour after LPS modeling. Control: as a blank control group; Model: intraperitoneal injection of LPS 100 *μ*g/kg; HLJDD L: HLJDD low dose group (1.58 g/kg); HLJDD M: HLJDD medium dose group (3.15 g/kg); HLJDD H: HLJDD high dose group (6.30 g/kg).

As shown in Figure 1, after LPS injection, the anal temperature increased in both model and HLJDD groups and reached the highest temperature at 6 h after LPS injection. The anal temperature of rats in each HLJDD treated group was lower than that of model group. The results showed that HLJDD had a certain antipyretic effect on LPS-induced rat fever.

### 3.2. Expression of Biochemical Indexes in Fever Rats

**Figure 2:**
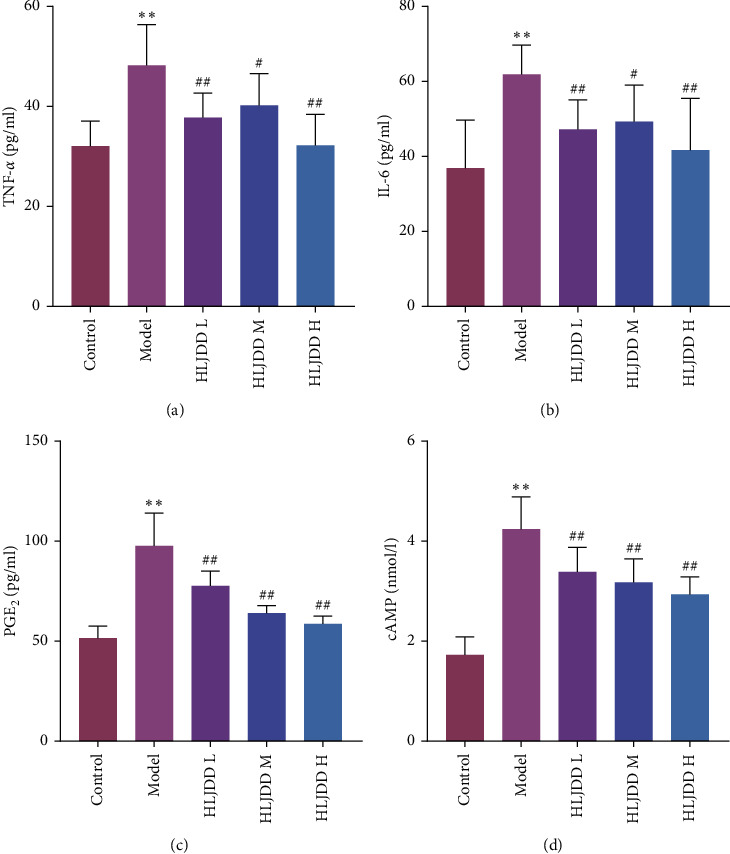
Effect of HLJDD on serum and hypothalamus tissue biochemical indexes of fever rats. (a-b) Expression of TNF-*α* and IL-6 in serum of fever rats. (c-d) Expression of PGE_2_ and cAMP in hypothalamus tissue of fever rats. ^*∗∗*^<0.01 versus control group. ^##^<0.01 versus model group, ^#^<0.05 versus model group. Control: as a blank control group; Model: intraperitoneal injection of LPS 100 *μ*g/kg; HLJDD L: HLJDD low dose group (1.58 g/kg); HLJDD M: HLJDD medium dose group (3.15 g/kg); HLJDD H: HLJDD high dose group (6.30 g/kg).

In order to explore the antipyretic effect of HLJDD on LPS-induced fever in rats, we detected the expression levels of TNF-*α* and IL-6 in the serum and PGE_2_ and cAMP in the hypothalamus tissue of fever rats (Figure 2). The expression of TNF-*α*, IL-6, PGE_2_, and cAMP in model group was significantly higher than that in control group (*P* < 0.01). It was shown that fever in the LPS-induced fever model in rats was closely associated with elevated levels of these indicators. Compared with model group, the expression of biochemical indexes in HLJDD intervention groups was significantly decreased (*P* < 0.05). Among them, HLJDD groups could significantly reduce the expression levels of PGE_2_ and cAMP in a concentration dependent manner (Figures 2(c) and 2(d)).

### 3.3. Prediction Results of Antipyretic Effect of HLJDD

#### 3.3.1. Compound-Target Network and Analysis

**Figure 3:**
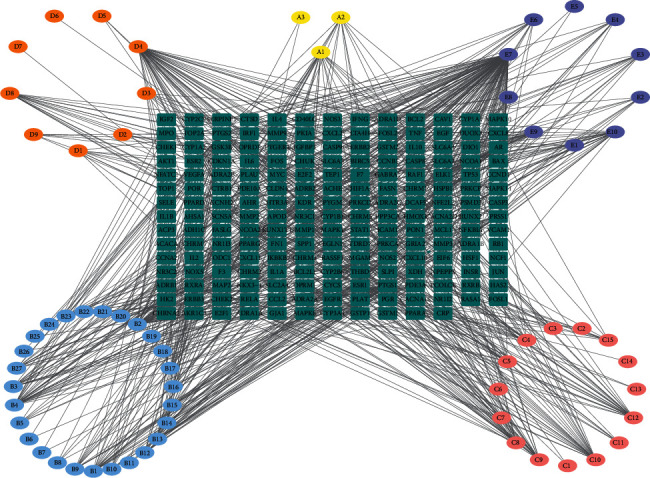
Herb-compound-target network of HLJDD (the ellipses represent components of HLJDD, the 3 yellow ellipses represent *Coptidis Rhizoma* components, the 27 blue ellipses represent *Scutellariae Radix* components, the 15 pink ellipses represent *Phellodendri Chinensis Cortex* components, the 9 orange ellipses represent *Gardeniae Fructus* components, the 10 purple ellipses represent the shared components in the four herbs of HLJDD, and the green round rectangles represent the 193 potential targets of HLJDD).

Due to the characteristics of multiple components and multiple targets, TCM compounds show a variety of pharmacological activities. Therefore, we constructed a network to study the potential mechanism of TCM compounds treating diseases. As shown in Figure 3, there are 257 nodes with 792 edges. Among these active components, we screened 20 components with high degree value, such as quercetin (MOL000098, degree = 127), kaempferol (MOL000422, degree = 50), wogonin (MOL000173, degree = 37), and baicalein (MOL002714, Degree = 29). These components with high degree value in the network are likely to be the main active components of HLJDD (Table 2).

#### 3.3.2. The PPI Network Construction of the Underlying Antipyretic Targets

**Figure 4:**
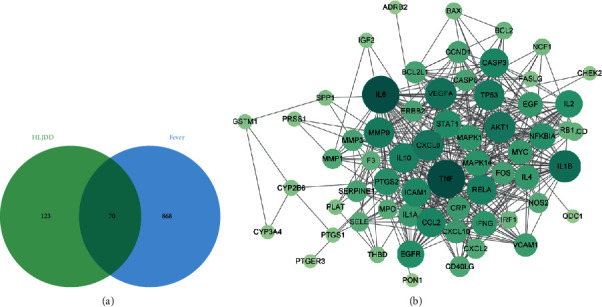
The network and analysis. (a) Distribution of HLJDD targets and the disease targets. (b) The PPI network. The higher the degree value is, the darker and larger the nodes in the graph will be.

A total of 64 chemical ingredients and 193 targets of HLJDD were collected and 939 targets of fever were obtained from GeneCard database (Figure 4(a)). These 70 intersection targets may be potential targets of antipyretic effect of HLJDD. The intersection targets were imported into STRING database. After that, PPI network of the underlying antipyretic targets was constructed by Cytoscape3.8.0 with 63 nodes and 455 edges (Figure 4(b)).

#### 3.3.3. GO Enrichment and KEGG Pathway Analysis

**Figure 5:**
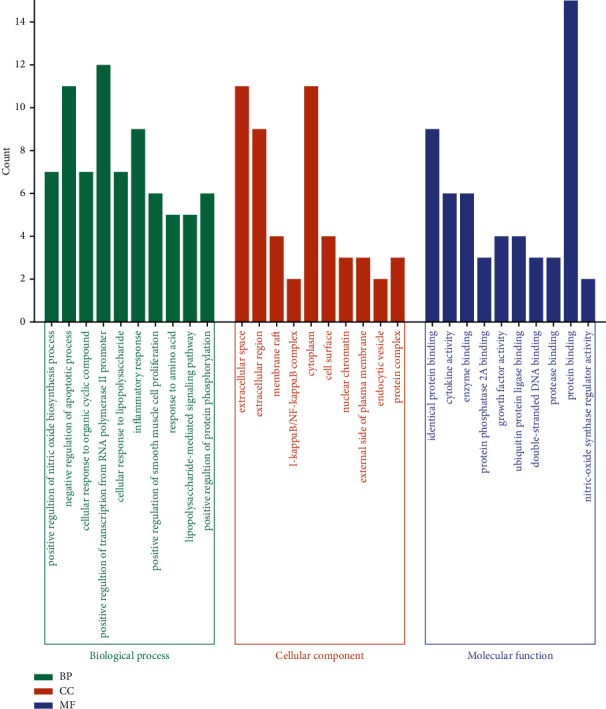
Top 10 GO terms of hub genes.

**Figure 6:**
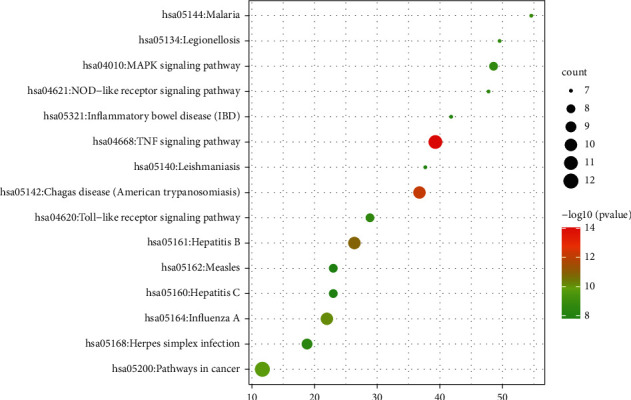
Top 15 KEGG pathway of hub genes.

The top 10 significantly enriched terms in biological process (BP), cellular component (CC), and molecular function (MF) categories are shown in Figure 5, which indicated that HLJDD may exert its antipyretic effect by regulating positive regulation of nitric oxide biosynthetic process, cellular response to organic cyclic compound, extracellular space, and identical protein binding. In order to explore the potential pathways involved in the antipyretic effect of HLJDD, we conducted KEGG pathway analysis, as shown in Figure 6, with 15 top signaling pathways. Among these 15 signaling pathways, MAPK signaling pathway plays a crucial role.

### 3.4. Experimental Verification on the Mechanism of HLJDD against LPS-Induced Fever

**Figure 7:**
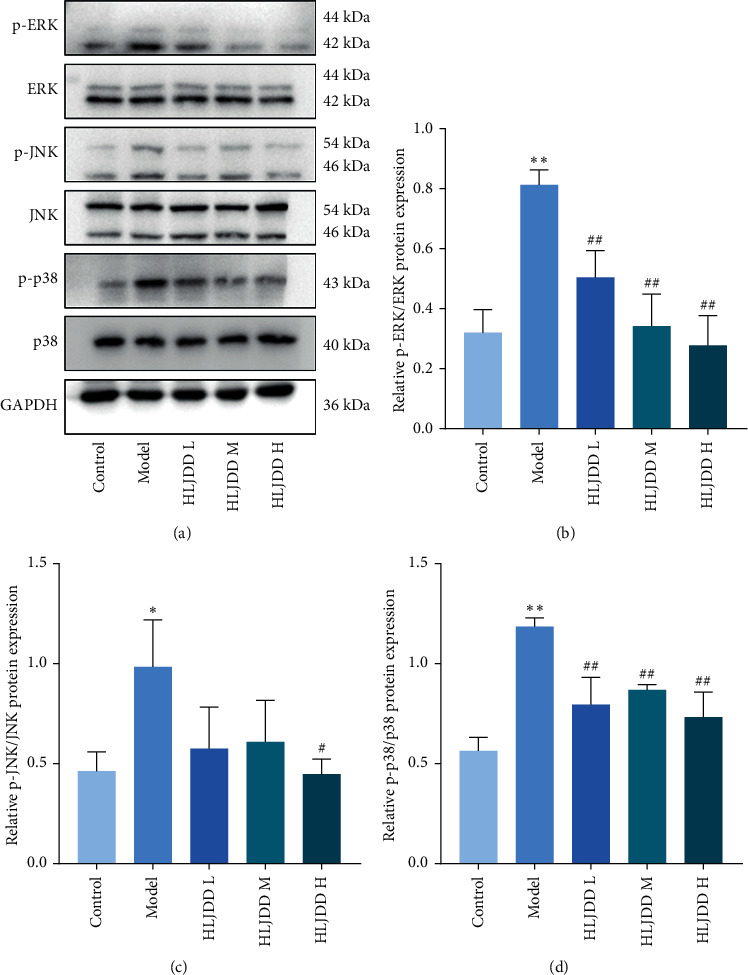
HLJDD suppresses the expression of MAPK signaling pathway. (a) Western Blotting images of ERK, p-ERK, JNK, p-JNK, P38, p-P38, and GAPDH. (b) Relative p-ERK protein expression in hypothalamus tissue. (c) Relative p-JNK protein expression in hypothalamus tissue. (d) Relative p-P38 protein expression in hypothalamus tissue. ^*∗∗*^<0.01 versus control group. ^##^<0.01 versus model group. ^*∗*^<0.05 versus control group. ^#^<0.05 versus model group. Control: as a blank control group; model: intraperitoneal injection of LPS 100 *μ*g/kg; HLJDD L: HLJDD low dose group (1.58 g/kg); HLJDD M: HLJDD medium dose group (3.15 g/kg); HLJDD H: HLJDD high dose group (6.30 g/kg).

To further evaluate the underlying antipyretic mechanism of HLJDD, MAPK signaling pathway was determined. As shown in Figure 7, the expression of ERK, p-ERK, JNK, p-JNK, P38, and p-P38 was detected and the results of Western Blotting were quantified by ImageJ. The results showed that, compared with the control group, the protein expression levels of p-ERK, p-JNK, and p-P38 were significantly increased after LPS injection (*P* < 0.05 or *P* < 0.01). HLJDD could downregulate their protein expression levels, which indicated that HLJDD has antipyretic effect on LPS-induced fever in rats through MAPK signaling pathway suppressing.

## 4. Discussion

Our study shows that HLJDD has a good antipyretic effect on LPS-induced fever in rats, and this effect may be carried out by inhibiting the MAPK signaling pathway.

HLJDD is a classic Chinese medicine prescription for clearing heat and detoxifying [28]. Previous studies have shown that HLJDD significantly reduces the levels of inflammatory factors such as IL-2, TNF-*α*, and IFN-*γ* and inflammatory mediators such as PGE_2_ and NO and suppresses immune and inflammatory responses [29, 30]. We studied the pharmacological effects of HLJDD (composed of *Coptidis Rhizoma*, *Scutellariae Radix*, *Phellodendri Chinensis Cortex*, and *Gardeniae Fructus* at 3 : 2 : 2 : 3) on LPS-induced fever in rats. At present, there are many methods to replicate the rat model of fever; the common ones are dry yeast, 2,4-dinitrophenol, and lipopolysaccharide. LPS, the outer membrane of Gram-negative bacteria, is a common febrifuge in animal experiments and stimulates macrophages and neutrophils to produce the endogenous pyrogens [31]. In this experiment, the rat fever model was established by intraperitoneal injection of LPS (100 *μ*g/kg). After the administration of HLJDD, it was found that different doses of HLJDD could reduce the temperature rise of rats, and the antipyretic effect was obvious. The biochemical indexes of inflammation were detected by enzyme-linked immunoassay kit, and HLJDD was found to reduce the secretion of LPS-induced inflammatory factors TNF-*α*, IL-6, PGE_2_, and cAMP. This finding suggests that HLJDD reduces body temperature, possibly by reducing proinflammatory cytokines and by inhibiting PGE_2_ and CAMP synthesis. This study is the first to investigate the antipyretic activity of HLJDD.

The multicomponent, multitarget nature of Chinese medicine makes its pharmacodynamic mechanism more complex. Therefore, with the help of modern science and technology, we combined network pharmacology to predict the main pharmacodynamic components of HLJDD, further predict the targets corresponding to the components, construct a component target network, and then screen the core targets and predict the possible pathways. Through network pharmacology analysis, we found that quercetin, kaempferol, wogonin, and baicalein might be the main pharmacodynamic components of HLJDD. Baicalin and its aglycone, baicalein, are the main components in *Scutellariae Radix*. The antioxidant and anti-inflammatory effects of baicalein have been demonstrated in a variety of disease models, including cardiovascular disease, inflammatory bowel disease, gout and rheumatoid arthritis, asthma, neurodegenerative diseases, liver and kidney diseases, and cancer [32]. For future research, we plan to select a compound from the prediction results of network pharmacology and explore the antipyretic effect and mechanism of a compound in HLJDD.

MAPK signaling pathway widely exists in all kinds of animal cells and participates in the regulation of cell proliferation, differentiation, transformation, and apoptosis through phosphorylated nuclear transcription factors, cytoskeleton proteins and enzymes, and is closely related to the occurrence of many diseases such as inflammation and tumor [33]. The MAPK pathway has four major branching routes, including ERK, JNK, p38/MAPK, and ERK5. Each MAPK signaling pathway has a relatively independent function. An important role of MAPK signaling pathway is to regulate cellular responses in response to changes in the extracellular environment. ERK pathway is mainly involved in cell proliferation and differentiation, while JNK pathway and p38MAPK pathway are mainly involved in cellular inflammatory response, stress response, and apoptosis [34]. Since MAPK signaling pathway is a classical inflammatory pathway, in addition, we referred to the predicted results of this network pharmacology and selected MAPK signaling pathway, using WB experiments as experimental validation. It was found that the administration of HLJDD reduced the expression levels of phosphorylated proteins of ERK, JNK, and p38 in different dose groups. In particular, the high dose group of HLJDD significantly inhibited the expression of related proteins in MAPK signaling pathway. In summary, HLJDD inhibits the protein expression level in MAPK signaling pathway, thus playing the role of antipyretic and relieving inflammation.

## 5. Conclusion

In this study, we investigated the antipyretic effects of HLJDD at the overall animal level by constructing a febrile rat model and combined with network pharmacology techniques to detect the serum levels of the pyrogenic factors IL-6 and TNF-*α* and the biochemical indicators PGE_2_ and cAMP in the hypothalamus of the model animals. The expression of ERK, p-ERK, JNK, p-JNK, P38, and p-P38, which are related proteins of MAPK signaling pathway, was analyzed in the hypothalamus. The results of this study suggest that HLJDD has antipyretic effect on LPS-induced fever in rats, and its potential mechanism may be related to the inhibition of MAPK signaling pathway. This study lays a theoretical foundation for further study of HLJDD in the treatment of fever.

## Figures and Tables

**Table 1:** Antibodies information.

Antibodies	Dilution	Manufacturers	Cat. No.
Rabbit anti- Erk1/2	1 : 1000	Cell Signaling Technology	4695
Rabbit anti-p-Erk1/2	1 : 2000	Cell Signaling Technology	4370
Rabbit anti- JNK	1 : 1000	Cell Signaling Technology	9252
Rabbit anti- p-JNK	1 : 1000	Cell Signaling Technology	4668
Rabbit anti- p38	1 : 1000	Cell Signaling Technology	8690
Rabbit anti- p-p38	1 : 1000	Cell Signaling Technology	4511
Rabbit anti-GAPDH	1 : 10000	Proteintech	10494-1-AP
Goat anti-Rabbit IgG (H&L)	1 : 10000	ZENBIO	511203

**Table 2:** Information for candidate bioactive components of HLJDD.

Molecule ID	Molecule name	OB (%)	DL	Degree	Molecule structure	Herb
MOL000098	Quercetin	46.43	0.28	127	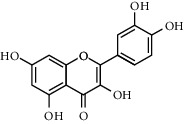	*Coptidis Rhizoma*, *Scutellariae Radix*, *Phellodendri Chinensis Cortex*, *Gardeniae Fructus*
MOL000422	Kaempferol	41.88	0.24	50	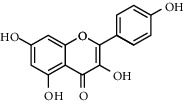	*Gardeniae Fructus*
MOL000173	Wogonin	30.68	0.23	37	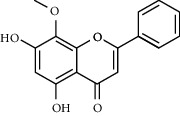	*Scutellariae Radix*
MOL002714	Baicalein	33.52	0.21	29	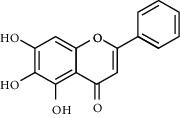	*Scutellariae Radix*
MOL000358	Beta-sitosterol	36.91	0.75	27	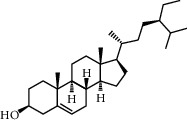	*Coptidis Rhizoma*, *Scutellariae Radix*, *Phellodendri Chinensis Cortex*, *Gardeniae Fructus*
MOL000790	Isocorypalmine	35.77	0.59	24	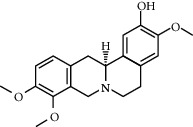	*Phellodendri Chinensis Cortex*
MOL000449	Stigmasterol	43.83	0.76	23	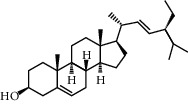	*Coptidis Rhizoma*, *Scutellariae Radix*, *Phellodendri Chinensis Cortex*, *Gardeniae Fructus*
MOL001455	(S)-Canadine	53.83	0.77	23	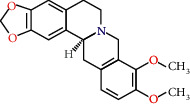	*Phellodendri Chinensis Cortex*
MOL002903	(R)-Canadine	55.37	0.77	22	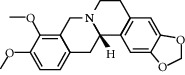	*Coptidis Rhizoma*
MOL002670	Cavidine	35.64	0.81	21	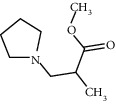	*Phellodendri Chinensis Cortex*
MOL000787	Fumarine	59.26	0.83	20	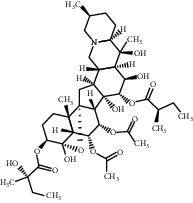	*Phellodendri Chinensis Cortex*
MOL002928	Oroxylin A	41.37	0.23	19	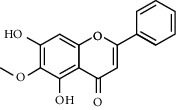	*Scutellariae Radix*
MOL001689	Acacetin	34.97	0.24	19	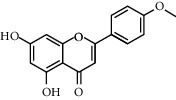	*Scutellariae Radix*
MOL003095	5-Hydroxy-7-methoxy-2-(3,4,5-trimethoxyphenyl)chromone	51.96	0.41	16	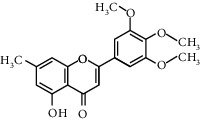	*Gardeniae Fructus*
MOL002651	Dihydrotanshinone II A	43.76	0.4	15	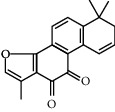	*Phellodendri Chinensis Cortex*
MOL008206	Moslosooflavone	44.09	0.25	15	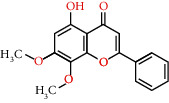	*Scutellariae Radix*
MOL000228	Alpinetin	55.23	0.2	15	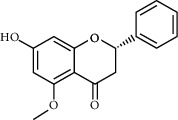	*Scutellariae Radix*
MOL002662	Rutaecarpine	40.3	0.6	14	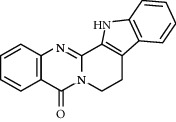	*Phellodendri Chinensis Cortex*
MOL002904	Berlambine	36.68	0.82	14	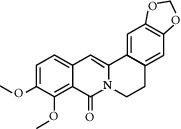	*Coptidis Rhizoma*
MOL000785	Palmatine	64.6	0.65	13	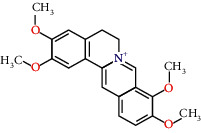	*Coptidis Rhizoma*, *Scutellariae Radix*, *Phellodendri Chinensis Cortex*, *Gardeniae Fructus*
MOL012266	Rivularin	37.94	0.37	13	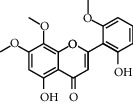	*Scutellariae Radix*
MOL002934	Neobaicalein	104.34	0.44	13	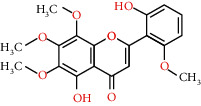	*Scutellariae Radix*
MOL001454	Berberine	36.86	0.78	12	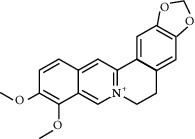	*Coptidis Rhizoma*, *Scutellariae Radix*, *Phellodendri Chinensis Cortex*, *Gardeniae Fructus*
MOL000552	5,2'-Dihydroxy-6,7,8-trimethoxyflavone	31.71	0.35	12	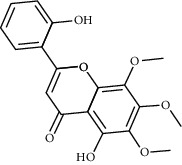	*Scutellariae Radix*
MOL002927	Skullcapflavone II	69.51	0.44	12	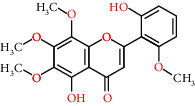	*Scutellariae Radix*

## Data Availability

The data used to support the findings of this study are available from the corresponding author upon reasonable request.
